# Nutritional Influences on Adiposity Rebound and Cardiometabolic Outcomes in a Prospective Birth Cohort of Low-Birth-Weight Children: A Study Protocol

**DOI:** 10.12688/wellcomeopenres.24589.1

**Published:** 2025-08-29

**Authors:** Neethu Thulaseedharan, Liss Maria Scaria, Srikant Ambatipudi, Deepa Bhaskaran, Sankar Hariharan, Akhila Sureshbabu, Panniyammakal Jeemon, Arun Gopalakrishnan

**Affiliations:** 1 Sree Chitra Tirunal Institute for Medical Sciences and Technology, Thiruvananthapuram, Kerala, 695011, India; 2 Child Development Centre, Thiruvananthapuram, Kerala, 695011, India; 3 Sree Avittam Tirunal hospital, Government Medical college, Thiruvananthapuram, Thiruvananthapuram, Kerala, 695011, India

**Keywords:** Low birth weight, adiposity gain, nutrition, cardiometabolic risk, cardiac structure, cardiac function, Neurodevelopment, DNA methylation

## Abstract

**Background:**

Nutritional practices during early life are critical in shaping long-term health outcomes. Poor or inappropriate nutrition may influence adiposity gain and the overall cardiometabolic risk among children born with low birth weight. Our study investigates how early feeding patterns, the timing of adiposity rebound, and DNA methylation of key genes influence cardiometabolic health at two years in low-birth-weight children.

**Methods:**

This study will be conducted as a longitudinal follow-up study among children with low birth weight (children born with a birth weight of less than 2500 grams). A thousand four hundred children will be recruited consecutively for this study. Birth weight, gestational age, and early neonatal and perinatal details will be collected from clinical records. Information on sociodemographic characteristics, dietary practices, and antenatal, obstetric, and postnatal histories will also be collected. The two-year follow-up assessment will include anthropometric measurements (height, weight, head circumference, chest circumference, waist circumference, and skinfold thickness) and blood pressure. Biochemical investigations will include a lipid profile, serum proteins, insulin resistance assessment, and hemoglobin levels. In addition, DNA methylation at six specific CpG sites relevant to adipogenesis and cardiometabolic health will be assessed. Left ventricular mass and ejection fraction will be evaluated using echocardiography. Carotid intima-media thickness will be measured using an appropriate ultrasound probe. The neurodevelopmental status of the children will be assessed using the Developmental Assessment Scales for Indian Infants (DASII) and Vineland Social Maturity Scale (VSMS).

**Conclusions:**

Elucidating the impact of early life nutritional practices is vital for promoting cardiometabolic health. This understanding supports the formulation of tailored feeding interventions that are essential for safeguarding cardiovascular health in children with low birth weight.

## Plain language summary

Nutritional Influences on Adiposity Rebound and Cardiometabolic Outcomes in a Prospective Birth Cohort of Low-Birth-Weight Children: A Study Protocol

Children born with low birth weight (LBW) are at a higher risk of developing excess body fat and heart and metabolic diseases later in life. Poor nutrition in early childhood may contribute to these risks. This study explores how feeding practices in early life, the timing of body fat changes (also known as adiposity rebound), and changes in the regulation of gene activity (measured through DNA methylation) influence long-term heart and metabolic health in LBW children.

We will follow 1,400 LBW children who are currently enrolled at the Child Development Centre (CDC) and have reached the age of two. The study is being led by the Sree Chitra Tirunal Institute for Medical Sciences and Technology (SCTIMST) in collaboration with the CDC. Researchers will collect detailed information about the children’s family and medical background, early nutrition, and growth using a specially designed questionnaire.

The study will also include physical measurements, blood tests, heart scans (echocardiography), ultrasound imaging of blood vessels (cIMT), analysis of DNA methylation patterns, and assessment of brain development. These tests will be performed at the SCTIMST and CDC.

By tracking how early life factors affect health as these children grow, this study aims to uncover key time periods in which interventions could help reduce the risk of future disease. Ultimately, this research will support efforts to design targeted strategies to improve the long-term health of children born with low birth weight.

## Introduction

The first thousand days of life, spanning from conception to two years of age, represent a critical period in human development ^1^. Adverse events during this window can increase susceptibility to chronic diseases later in life, forming the basis of the Developmental Origins of Health and Disease (DoHaD) hypothesis ^2^. This theory posits that early life environmental challenges trigger structural and functional adaptations that increase vulnerability to cardiometabolic disorders ^3^. Such prenatal and postnatal influences can disrupt physiological processes, increasing the likelihood of early adiposity or premature adiposity rebound ^2, 3^. Typically, adiposity rebound occurs between the ages of five and seven in children with normal birth weight ^4^. However, adiposity rebound before the age five is classified as early adiposity rebound ^5^, a risk factor for future cardiovascular diseases, is more prevalent among children born with low birth weight (LBW).

Diverse factors, such as distinct dietary patterns and interactions between genes and the environment, could increase the likelihood of LBW children to develop early adiposity rebound. Adiposity measurements among children with LBW are closely linked to DNA methylation patterns in specific obesity-related genes. This could be due to prenatal programming, postnatal overnutrition or undernutrition, and clinically significant adiposity gain in children with LBW ^6^. DNA methylation may also mediate the association between childhood adiposity and future cardiometabolic risk ^7^. The relationship between early feeding practices, adiposity patterns, and cardiometabolic consequences among low-birthweight children living in Indian settings has not been well explored. This stems from the lack of well-characterized birth cohorts of LBW children with a detailed assessment of nutritional patterns and adverse consequences during the first thousand days of life, which limits the exploration of adiposity patterns and their cardiometabolic consequences in India. Additionally, the impact of early life dietary patterns on epigenetic pathways that contribute to early adiposity gain or adiposity rebound remains underexplored.

Despite Kerala’s advanced epidemiological transition, low birth weight remains a significant public health concern with a prevalence of 11.3% ^8^. Establishing a well-characterized LBW cohort in the state provides a valuable opportunity to investigate the relationships between early feeding practices, adiposity gain, and subsequent cardiometabolic outcomes. Early identification and risk stratification within the first thousand days can reveal critical intervention points to mitigate cardiometabolic risk progression in children with LBW. Accordingly, this study aim to conduct a comprehensive follow-up of an LBW cohort at two years of age, examining the influence of early nutrition and DNA methylation on adipogenesis and cardiometabolic health, along with the assessment of growth and developmental trajectories.

## Methods

### Ethical considerations

The study is approved by the Institutional Ethics Committee of the Sree Chitra Tirunal Institute for Medical Sciences and Technology (approval no. SCT/IEC/2023/May/2023, dated 19-09-2023) and the Child Development Centre (Approval No. 03/CDC/2021, dated 29-12-2021).

All data collected from study participants will be handled with strict confidentiality. No personally identifiable information will be published or disclosed. Participants will be assigned unique identification codes and names or other personal identifiers will not be recorded in the electronic database.

Written informed consent will be obtained from the parents or legal guardians prior to data collection. The consent process will include a clear explanation, in the local language, of the study’s purpose, procedures, and the nature of the assessments and investigations involved. Participation in the study will be voluntary. Parents will be informed that they may choose not to answer specific questions or withdraw their child from the study at any point, without any consequences.

While the procedures are generally non-invasive, blood collection may cause minor discomfort. A trained healthcare professional collects approximately 5 ml of blood using standard safety protocols to ensure minimal risk and discomfort. Parents will receive a copy of the test results from blood investigations. The participant information sheet also emphasizes the confidentiality of all shared data and reiterate that no identifying information would be used in the study outputs.

### Study design and setting

The study is designed as a longitudinal follow-up study. It will be conducted at the Child Development Centre (CDC) and the Sree Chitra Tirunal Institute for Medical Sciences and Technology (SCTIMST), Kerala. LBW children admitted to the Neonatal Intensive Care Units of a government tertiary care hospital in the Thiruvananthapuram district of Kerala are directly referred to the CDC after discharge, and they are under follow-up from birth to two years of life. During routine visits, a team consisting of a developmental pediatrician, developmental therapist, and clinical psychologist assess their physical, psychological, behavioral, neurodevelopmental, and cognitive status to detect health or developmental problems. We will conduct a detailed clinical follow-up at the two years of age.

### Study population

Children with LBW, registered in the CDC from 2021 to 2024 will be recruited for this study. The current study will be conducted among LBW children who attain two years between 2024–2027. Children whose parents did not consent to participate and those who did not have at least three routine follow-ups visit will be excluded.

### Sample size and sampling

The sample size was calculated using OpenEPI, version 3.1. As per the New Delhi birth cohort, the proportion of impaired glucose tolerance among exposed children (children with adiposity rebound at 2–5 years) was 21%. Similarly, the percentage of unexposed (without rebound adiposity) children with the outcome (metabolic risk) was 15 percent ^9^. We calculated the sample size as 1140 based on a 95% confidence level and 80% power. Assuming an attrition rate of 20%, the sample size was increased to 1400. Echocardiogram and methylation studies will be conducted among all children with adiposity gain/rebound and a sub-sample of 200 children without adiposity gain or rebound.

### Data collection

The outcomes of this study are cardiometabolic risk, cardiac structure and function, and neurodevelopmental status of children. The measurement details of the outcome variables are listed in Table 1.

**Table 1.** Study outcomes.

Outcome	Measurement
Cardiometabolic risk	Blood pressure, insulin resistance, dyslipidemia, and carotid Intima Media Thickness (cIMT) will be assessed as part of cardiometabolic risk in the study.
Cardiac structure and function	The cardiac structure and function of the children will be determined by the assessment of Left Ventricular Mass (LVM) and left ventricular ejection fraction.
Neurodevelopment	The neurodevelopmental status of the children will be assessed using the Developmental Assessment Scales for Indian Infants (DASII) ^10^ and the Vineland Social Maturity Scale (VSMS).

Blood pressure (BP) will be assessed using an Omron (model: BSX515) digital BP apparatus with a cuff size of 10–19 cm. Blood pressure includes systolic and diastolic blood pressure. The BP will be measured after the child sits to rest for 5 min. Two readings will be taken at 15-minute intervals, and the average of these two readings will be considered. Blood pressure based on sex, age, and height table in “The Fourth Report on the Diagnosis, Evaluation, and Treatment of High Blood Pressure in Children and Adolescents” will be used as a reference standard. A BP of < 90th percentile will be considered normal. Blood pressure between the 90 ^th^ and 95 ^th^ percentiles is considered as prehypertension, and blood pressure greater than the 95 ^th^ percentile will be regarded as hypertension ^10^.

Basic biochemical blood parameters will be assessed, including insulin resistance, lipid profiles, serum proteins, and hemoglobin levels. Insulin resistance will be evaluated based on the Homeostatic Model Assessment for insulin resistance (HOMA-IR) (fasting insulin (mg/dl) × fasting glucose (µU/ml) /22.5. An HOMA-IR greater than 2.5 will be considered to indicate insulin resistance ^11^. The insulin will be analyzed using a Chemiluminescence Analyzer (Cobas e411) (Roche Diagnostics). Lipid profiles, including total cholesterol, High-Density Lipoprotein (HDL) cholesterol, Low-Density Lipoprotein (LDL) cholesterol, and triglycerides, will be assessed using EM360- Erba – Tranasia Bio-Medicals Limited. A total cholesterol level of less than 170 mg/dl is optimal; 170–199 is borderline high, and 200 or more is high. An LDL value of less than 110 is considered optimal, 110–129 is borderline high, and a value of 130 or more is high. Triglyceride levels less than 75 mg/dl are considered optimal, 75–99 mg/dl is borderline, and 100 mg/dl or more is high. An HDL cholesterol level of more than 45 mg/dl is considered optimal, and less than 40 mg/dl is considered low ^11^. Serum proteins, including albumin, globulin, albumin to globulin ratio, and total proteins, will also be assessed in EM360-Erba – Tranasia Bio Medicals Limited. Hemoglobin will be analyzed using Mindray BC 6800. Hemoglobin levels below 11 g/dl is considered anemic ^12^.

The carotid intima-medial thickness (cIMT) will be assessed by a consultant pediatric cardiologist when the child is cooperative and awake or sleeping without the use of any sedative. The cIMT will be evaluated using the Esaote My Lab Gamma Ultrasound machine with a linear array transducer 3–13 MHz SL1543. Longitudinal 2D images of the common carotid artery will be obtained and zoomed live to display the adventitia and intima as echogenic lines separated by hypoechoic media. The far wall of the distal common carotid artery will be imaged at least 5 mm proximal to bifurcation. The distance between the leading edge of the lumen-intima interface and the leading edge of the media-adventitia interface will be measured using electronic calipers to obtain cIMT. cIMT will be measured (in millimeters) with the anterolateral position ^13^. Three readings will be obtained from each left and right common carotid artery, and their average will be considered for analysis.

Cardiac structure and function will be ascertained using transthoracic echocardiography. Echocardiography will be performed by a consultant pediatric cardiologist in a quiet room when the child is awake or sleeping without using any sedative medication. The recommendations of the American Society of Cardiology will be followed for ECHO assessment ^14– 16^. Echocardiography will be conducted using a Philips EPIQ 7C echocardiography machine with a pediatric high-frequency probe S8-3. Left ventricular mass and ejection fraction will be assessed using echocardiography. Left ventricular mass assessment will be conducted through left ventricular M-mode tracing, which will be evaluated using the parasternal Long Axis (PLAX) and parasternal short axis views (PSAX) ^17^. The left interventricular end-diastolic septal thickness (IVSTD), left ventricular end-diastolic diameter (LVEDD), and left ventricular end-diastolic posterior wall thickness (LVPWTD) will also assessed. Left ventricular mass (grams) computed using the formula derived by Devereux *et al*. ^18^.

LV mass = 0.80 × 1.04((IVSTD + LVEDD + LVPWTD)3 - (LVEDD)3) + 0.6

The left ventricular ejection fraction (in percentage) will be assessed as a part of cardiac function. It will be evaluated by planimetry using Simpson’s biplane method. The endocardial left ventricular border in end-diastole (LVED) and the left ventricular border in end-systole (LVES) will be traced from the apical four-chamber and two-chamber windows to obtain the end-diastolic and end-systolic volumes by biplane analysis.

Ejection fraction (%) = (End-diastolic volume - End-systolic volume) / end-diastolic volume × 100.

The developmental status of children will be assessed using the Developmental Assessment Scales for Indian Infants (DASII) ^10^ and Vineland Social Maturity Scale (VSMS) ^19^. The DASII test is conducted for infants from birth and 2.5 years of age. It comprises motor and mental clusters. Motor clusters have 67 items, and mental clusters have 163 items. The motor and mental age and motor and mental development quotients will be calculated using the DASII. In addition, the score and percentile for each cluster will also be identified. The VSMS scale measures the differential social capacity of an individual. It estimates social age and social quotient, and is highly correlated with intelligence. It is designed to measure social maturation in eight social areas: self-help general, self-help eating, self-help dressing, Self-direction, Occupation, Communication, Locomotion, and Socialization ^19^.

Other exposure factors include sociodemographic factors, maternal health factors (maternal health and demographics), prenatal exposures and complications during pregnancy, birth, and neonatal, epigenetic factors, nutritional factors, growth, and adiposity. Sociodemographic information, antenatal history, obstetric details, and neonatal details will be collected at the time of follow-up at two years and from clinical records.

Information on the nutritional practices of the children during the two years of life will be ascertained using a structured questionnaire. Feeding practices during the initial days of life will be explored, including breastfeeding practices (exclusive breastfeeding and breastfeeding along with complementary feeding), formula-feeding practices, and initiation of weaning. Current food habits will also be collected, including regular meals (number of main meals and snacks per day), water intake, and consumption of vegetables and fruits, dairy products, meat products, and processed foods.

The growth and adiposity of the children will be identified through anthropometric measurements, including height (measured using HSCO stadiometer: 7045922204), weight (assessed using Equal digital weighing machine, model: EQPW-003), head circumference, waist circumference, mid-upper arm circumference (MUAC) (measured using non-stretchable measuring tape), and skinfold thickness (triceps and subcapsular skin fold thickness will be assessed using Cescorf stainless steel skinfold calipers). The World Health Organization (WHO) child growth standards will be followed for the growth assessment of children ^20^. The Centers for Disease Control (CDC) anthropometric measurement manual will be used as a reference for measurement ^21^.

We will assess the DNA methylation of specific genes. DNA methylation is one of the most studied epigenetic modifications, in which methyl group is added to the C5 position of cytosine forms 5-methylcytosine. DNA methylation is known to regulate expression of gene ^22^. The CpG (5'—C—phosphate—G—3') sites at the promoter region of selected genes previously reported to be associated with cardiometabolic risk—ABCG1 (ATP binding cassette subfamily G member 1), CPT1A (carnitine palmitoyl transferase 1A), SLC6A4 (solute carrier family 6 member 4), GPR120 (G protein–coupled receptor 120), FABP5 (fatty acid binding protein 5), and CHGA (Chromogranin A)—will be included for DNA methylation analysis. Methylation of ABCG1 and CHGA was associated with glucose metabolism ^23, 24^, whereas CPT1A, FABP5, and GPR120 are linked to lipid metabolism ^24^ and SLC6A4 is associated with adiposity ^25^. The blood sample for the DNA methylation study will be collected in an EDTA vacutainer. DNA will be extracted from whole blood using a Macherey-Nagel Nucleospin DNA Extraction Kit (740951.10/.50/250). The quantity and quality of extracted DNA will be assessed using a NanoDrop spectrophotometer and by running agarose gel respectively. Assessment of DNA methylation at specific CpG sites will be performed using methylight assays ^26^. We will follow a two-step process. First, the extracted DNA will be modified by treating it with sodium bisulphite, which converts unmethylated cytosines to uracil. The methylated cytosine will remain unchanged during this process. The bisulphite-treated DNA will undergo two separate reactions: one with the methylated primer set and the other with the unmethylated primer set. Subsequently, it will be amplified using Polymerase Chain Reaction (PCR) according to the manufacturer’s protocol.

An overview of the follow-up assessments of the children is presented in Table 2.

**Table 2.** Overview of child and parental follow-up and assessments.

Time points of child and parent follow-up							
From clinical records							Follow-up clinic visits
Domain	Type of assessment	At birth	1 M	3 M	6 M	1 Year	2 Years
Sociodemographic information, obstetric and natal information	Baseline questionnaire	√					√
Growth and development	Height	√	√	√	√	√	√
	Weight	√	√	√	√	√	√
	Head circumference	√	√	√	√	√	√
	Chest Circumference						√
	Waist circumference						√
	Mid Upper Arm Circumference						√
	Skinfold thickness						√
	Development Assessment Scale for Indian Infants						√
	Vineland Social Maturity Scale						√
Blood pressure							√
Diet	Breastfeeding practices	√	√	√	√	√	√
	Dietary habits or patterns				√	√	√
Blood investigations	Insulin						
	Glucose						√
	Lipid profile						√
	Hemoglobin and blood routine						√
	S proteins						√
	DNA methylation analysis						√
Echocardiography							√
Carotid Intima Media Thickness							√

√=Time points of child follow-up and assessment DNA: Deoxyribonucleic acid

### Data analysis

All study data will be collected and managed using Research Electronic Data Capture (REDCap), a secure web-based software platform designed to support data capture for research studies. Data analysis will be conducted using SPSS version 26.0, R (version 4.0 or higher), and STATA version 16.

The cumulative incidence of adiposity gain or rebound among the cohort will be calculated to determine the proportion of children experiencing early adiposity gain or rebound by the age of two. To compare changes in continuous variables (e.g., anthropometric or biochemical measures) between the baseline and follow-up, paired t-tests will be used. Categorical variables (e.g., feeding practices or presence of clinical conditions) will be compared using Pearson’s chi-square test.

To evaluate the cardiometabolic risk associated with early adiposity gain or adiposity rebound, relative risks (RR) and their corresponding 95% confidence intervals will be estimated by comparing children with early adiposity gain or adiposity rebound to those without.

Longitudinal relationships and potential causal pathways linking early life exposures (e.g., feeding patterns and DNA methylation status) to outcomes related to growth, neurodevelopment, and cardiometabolic health will be assessed using linear and nonlinear mixed-effects regression models, accounting for repeated measures and potential confounders.

In addition, risk ratios (RR) and odds ratios (OR) will be calculated to examine associations between early life determinants and cardiac structure and function, as well as markers of metabolic risk. All statistical tests will be two-tailed, and a p-value <0.05. will be considered statistically significant. Estimates will be reported with 95% confidence intervals.

### Dissemination

The results of this study will be disseminated through publications, newspaper reports, and social media outlets. In addition, the major findings will be communicated to key stakeholders, health care providers, and policymakers. The results will be presented at national and international conferences.

## Discussion/Conclusions

Low birth weight (LBW) continues to be a significant public health challenge, particularly in low- and middle-income countries such as India, where it poses serious risks to both maternal and child health ^27^. To our knowledge, this is the first prospective birth cohort study in India specifically designed to investigate the long-term health outcomes of LBW children. Previous research has predominantly focused on children with normal birth weight, exploring their nutritional needs and health trajectories into adulthood ^28– 31^. However, comprehensive data on LBW children within the Indian context remain scarce. Importantly, findings from studies on normal-birth-weight children may not be generalizable to LBW populations because of distinct physiological and metabolic differences that influence their development and health outcomes ^32– 34^.

It is essential to understand early-life feeding practices and their effects on growth, development, and cardiometabolic health in children with LBW. This study comprehensively investigates the influence of nutritional patterns during infancy and early childhood on long-term cardiometabolic outcomes. A key focus is the timing of adiposity rebound or early adiposity gain and its potential role in shaping future cardiometabolic risk. Additionally, this study explores the underlying epigenetic mechanisms, particularly the role of DNA methylation, in mediating the relationship between early adiposity trajectories and later cardiometabolic health.

The longitudinal design of this study facilitates the ongoing follow-up of the cohort across various developmental stages, allowing for a detailed examination of health trajectories over time. This approach enables the identification of critical windows of vulnerability as well as opportunities for targeted prevention and early intervention. By elucidating the influence of early life factors on cardiometabolic health, this study enhances our understanding of long-term health risks in LBW children. Furthermore, the insights gained support the development of customized interventions aimed at addressing the specific health and developmental needs of this high-risk population.

In summary, this longitudinal study fills a critical gap by investigating early nutrition, adiposity rebound timing, and DNA methylation in low-birth-weight Indian children and elucidating their impact on cardiometabolic outcomes. It identifies key developmental windows for intervention and, inform targeted strategies to mitigate long-term health risks in this vulnerable population.

## Data Availability

No underlying data are associated with this article. The protocol’s STROBE checklist is available at https://doi.org/10.6084/m9.figshare.29629754.v1 ^35^. Questionnaire used in the study is available at https://doi.org/10.6084/m9.figshare.29611073.v1 ^36^. and the information sheet and consent form is available at https://doi.org/10.6084/m9.figshare.29611055.v1 ^37^. Data are available under the terms of the Creative Commons Attribution 4.0 International license (CC-BY 4.0).
