# Supplementary material for: Nutritional Influences on Adiposity Rebound and Cardiometabolic Outcomes in a Prospective Birth Cohort of Low-Birth-Weight Children: A Study Protocol
Source: Wellcome Open Res. 2025 Dec 8;10:473. Originally published 2025 Aug 29. [Version 2] doi: 10.12688/wellcomeopenres.24589.2 (PMC12550497; doi:10.12688/wellcomeopenres.24589.2)
Supplement: Peer review history

# Reviewer report: Reviewer response for version 2

**DOI:** 10.21956/wellcomeopenres.28165.r141843

**Published:** 2025-12-17

**Authors:** Giridhara Rathnaiah Babu

**Affiliations:** 1 Qatar University, Doha, Qatar

All comments hae been addressed. The manuscript is okay to be indexed.

Is the study design appropriate for the research question?

Yes

Is the rationale for, and objectives of, the study clearly described?

Partly

Are sufficient details of the methods provided to allow replication by others?

Yes

Are the datasets clearly presented in a useable and accessible format?

Yes

Reviewer Expertise:

Cohort, Lifecourse epidemiology

I confirm that I have read this submission and believe that I have an appropriate level of expertise to confirm that it is of an acceptable scientific standard.

# Reviewer report: Reviewer response for version 1

**DOI:** 10.21956/wellcomeopenres.27098.r134682

**Published:** 2025-10-23

**Authors:** GV Krishnaveni

**Affiliations:** 1 CSI Holdsworth Memorial Hospital, Mysore, Karnataka, India

This manuscript describes a study protocol to explore the relationship between early adiposity rebound and cardiometabolic outcomes in a cohort of low-birth weight children. The study proposes to make use of the available low birthweight registry to recruit a large sample of children at two years of age. The researchers plan a wide range of assessments including anthropometry, blood parameters and cardiac parameters, and epigenetic analysis. This is an ambitious study and the details are well- presented. I have a few questions for better understanding of the study premise.  

1. Early adiposity rebound is more common in large babies. DOHaD hypothesis posits that those born with lower birthweights (a proxy for fetal growth deficits) are likely to grow adipose relative to themselves   Any other evidence related to small-for gestation babies and early adiposity rebound? Background section may benefit with additional information on this association.
2. As the children are studied exactly at two years of age, what is the likelihood of detecting adiposity rebound as it may occur any time before the age of five?  
3. Considering the above issue, the researchers may use continuous anthropometric variables,  in addition to adiposity rebound (yes/no) in their analysis plan.  

Is the study design appropriate for the research question?

Yes

Is the rationale for, and objectives of, the study clearly described?

Yes

Are sufficient details of the methods provided to allow replication by others?

Yes

Are the datasets clearly presented in a useable and accessible format?

Not applicable

Reviewer Expertise:

Epidemiological scientist with ~three decades of experience in long-term cohort studies. My research interests relate to the developmental origins of adult non-communicable diseases.

I confirm that I have read this submission and believe that I have an appropriate level of expertise to confirm that it is of an acceptable scientific standard.

# Response

**Published:** 2025-12-04

**Authors:** Panniyammakal Jeemon

**Affiliations:** Sree Chitra Tirunal Institute for Medical Sciences and Technology, Trivandrum, India

**Comment:** Early adiposity rebound is more common in large babies. DOHaD hypothesis posits that those born with lower birthweights (a proxy for fetal growth deficits) are likely to grow adipose relative to themselves. Any other evidence related to small-for gestation babies and early adiposity rebound? Background section may benefit with additional information on this association.

**Response**: Evidence from several longitudinal studies indicates that Low Birth Weight children or children born Small for Gestational Age (SGA) have increased risk for developing an earlier adiposity rebound. This increased risk may largely be mediated by the rapid postnatal catch-up growths and changes in adiposity distribution and alterations in insulin or Insulin like Growth Factor (IGF) signalling. This information has been added to the revised manuscript.

**Comment:** As the children are studied exactly at two years of age, what is the likelihood of detecting adiposity rebound as it may occur any time before the age of five?  Considering the above issue, the researchers may use continuous anthropometric variables, in addition to adiposity rebound (yes/no) in their analysis plan.  

**Response:**  Since adiposity rebound can occur at any time before the age of five, we will assess the incidence of adiposity rebound or adiposity gain in this cohort at two years of age. Continuous anthropometric data will be used to evaluate patterns of adiposity gain.

# Reviewer report: Reviewer response for version 1

**DOI:** 10.21956/wellcomeopenres.27098.r132312

**Published:** 2025-10-13

**Authors:** Giridhara Rathnaiah Babu

**Affiliations:** 1 Qatar University, Doha, Qatar

This is an important study.  I have a few concerns/suggestions.

- The rationale for performing echo and carotid intima thickness assessments at 2-year follow-up is unclear. Specifically, since anthropometric markers are assessed, what is the need to perform an Echocardiogram in all children? What is the biological basis of the early development of cardiac abnormalities?  
- The researchers want to assess cardiometabolic markers. Yet, adiposity assessments using standard techniques not mentioned (Air Displacement Plethysmography, or other methods)
- Are there any plans to store any samples? Is there a biorepository ? Have ethical permissions been obtained for it?
- A directed acyclic graph (DAG) specifying the study variables (exposure, outcome, and confounders) along with their specific time points is required to justify the hypothesis. 

Is the study design appropriate for the research question?

Yes

Is the rationale for, and objectives of, the study clearly described?

Partly

Are sufficient details of the methods provided to allow replication by others?

Yes

Are the datasets clearly presented in a useable and accessible format?

Yes

Reviewer Expertise:

Cohort, Lifecourse epidemiology

I confirm that I have read this submission and believe that I have an appropriate level of expertise to confirm that it is of an acceptable scientific standard.

# Response

**Published:** 2025-12-04

**Authors:** Panniyammakal Jeemon

**Affiliations:** Sree Chitra Tirunal Institute for Medical Sciences and Technology, Trivandrum, India

**Comment:  **The rationale for performing echo and carotid intima thickness assessments at 2-year follow-up is unclear. Specifically, since anthropometric markers are assessed, what is the need to perform an Echocardiogram in all children? What is the biological basis of the early development of cardiac abnormalities?  

**Response: ** Thank you for your comment regarding the rationale for including echocardiogram and carotid intima-media thickness (cIMT) assessments at the 2-year follow-up. While anthropometric measures provide valuable information on growth and adiposity, they do not capture early, subclinical cardiovascular changes that may originate in childhood. Adverse intrauterine exposures can induce permanent structural and functional changes in the heart, as demonstrated in prior studies (1) Foetal growth has also been linked to differences in cardiac volume and mass, suggesting that early alterations may serve as predictors for future cardiometabolic risk (2). ). Furthermore, increased cIMT is recognized as a surrogate marker of cardiovascular risk (3), and children born small for gestational age (SGA) have been shown to exhibit higher cIMT values (4). Assessing both echocardiographic and vascular parameters alongside anthropometry therefore provides a more comprehensive evaluation of early cardiovascular health.

**Comment:** The researchers want to assess cardiometabolic markers. Yet, adiposity assessments using standard techniques not mentioned (Air Displacement Plethysmography, or other methods)

**Response: **Standard adiposity assessment methods like Air Displacement Plethysmography (ADP) are indeed widely used for older children; however, their application in children under 6 years presents notable challenges. The accuracy of ADP in very young children is limited by several factors, including the small body size, behavioral immaturity, and the difficulty of maintaining stillness during the measurement, all of which can introduce substantial error into the results. Additionally, young participants often exhibit movement, crying, or separation anxiety when placed alone in the device, further compromising measurement reliability or leading to high rates of technical failure (5). . Given that our study subjects are two years old, these practical and technical limitations make ADP an unsuitable method for this age group in our research context (6)

**Comment:** Are there any plans to store any samples? Is there a biorepository? Have ethical permissions been obtained for it?

**Response: **The samples will be stored for future research in a -80-degree deep freezer. The permission has been obtained from IEC of the institution.

**Comment:** A directed acyclic graph (DAG) specifying the study variables (exposure, outcome, and confounders) along with their specific time points is required to justify the hypothesis. **Response:** We have added the diagram to the main manuscript.

 References

 1.         Crispi F, Hernandez-Andrade E, Pelsers MMAL, Plasencia W, Benavides-Serralde JA, Eixarch E, et al. Cardiac dysfunction and cell damage across clinical stages of severity in growth-restricted fetuses. Am J Obstet Gynecol. 2008 Sept;199(3):254.e1-8.

 2.         Nikolaidou C, Karamitsos T. The shape of our hearts: The impact of early stages in life on cardiac development. Eur J Prev Cardiol. 2020 Jan 1;27(1):60–2.

 3.         Willeit P, Tschiderer L, Allara E, Reuber K, Seekircher L, Gao L, et al. Carotid Intima-Media Thickness Progression as Surrogate Marker for Cardiovascular Risk: Meta-Analysis of 119 Clinical Trials Involving 100 667 Patients. Circulation. 2020 Aug 18;142(7):621–42.

 4.         Epure AM, Rios-Leyvraz M, Anker D, Di Bernardo S, da Costa BR, Chiolero A, et al. Risk factors during first 1,000 days of life for carotid intima-media thickness in infants, children, and adolescents: A systematic review with meta-analyses. PLoS Med. 2020 Nov 23;17(11):e1003414.

 5.         Air‐Displacement Plethysmography Pediatric Option in 2–6 Years Old Using the Four‐Compartment Model as a Criterion Method - Fields - 2012 - Obesity - Wiley Online Library [Internet]. [cited 2025 Oct 17]. Available from: https://onlinelibrary.wiley.com/doi/full/10.1038/oby.2012.28

 6.         Bijlsma A, van Beijsterveldt IALP, Vermeulen MJ, Beunders VAA, Dorrepaal DJ, Boeters SCM, et al. Challenges in body composition assessment using air-displacement plethysmography by BOD POD in pediatric and young adult patients. Clin Nutr. 2023 Sept 1;42(9):1588–94.
